# A Study on a Polymeric Foam Based on Pulse Proteins and Cellulose Fibrils

**DOI:** 10.3390/ma16144965

**Published:** 2023-07-12

**Authors:** Marcela Jarpa-Parra, Sergio Moraga-Bustos, Eduardo Gutiérrez-Turner, Gipsy Tabilo-Munizaga

**Affiliations:** 1 Núcleo de Investigación en Agroalimentos y Nutrición Aplicada, Universidad Adventista de Chile, Chillán 3780000, Chile; 2 Facultad de Ingeniería, Universidad Adventista de Chile, Chillán 3780000, Chile; sergiomoraga@unach.cl; 3 Instituto de Estadística, Universidad de Valparaíso, Valparaíso 2340000, Chile; eduardogutierrez@unach.cl; 4 Facultad de Educación, Universidad Adventista de Chile, Chillán 3780000, Chile; 5 Food Engineering Department, Universidad del Bío-Bío, Av. Andrés Bello 720, Chillán 3780000, Chile; gtabilo@ubiobio.cl

**Keywords:** cellulose fibrils, lentil protein, biofoam, rheology, microstructure

## Abstract

Biofoams are a challenge for scientists in terms of innovation. Incorporation of cellulose fibrils (CF), might help improve the microstructure of foams, thus this study focuses on studying the impact of CF on the foaming properties and rheology of lentil protein (LP) foams at various pH and CF concentrations. Additionally, LP-CF mixtures were transformed into solid foams, and their microstructure, physical properties, and morphology were evaluated. CF concentration significantly impacted on LP-CF foam properties, primarily due to high viscosity values. Increased CF concentration resulted in improved FS values (up to 77 min) at all pH values. This is likely attributed to associative interactions and coacervates formation. Also, foam microstructure could be related to apparent viscosity, suggesting the role of viscosity in preserving the integrity of the wet foam structure during freezing and lyophilization processes. However, elevated viscosity values might negatively impact properties such as foaming capacity and produce denser microstructures. The microstructure and morphology analysis revealed that certain foams exhibited a sponge-like structure with open pores and semi-spherical shapes, supported by CF fibers extending and forming layers. However, the structure itself was irregular. While others exhibited non-uniform, irregular pore size, and shape, along with a denser structure. These findings contribute to understanding the behavior of LP-CF mixtures, although additional investigations on mechanical properties, biodegradability, and hydrophobicity are necessary to reach their full potential for various applications.

## 1. Introduction

Foams are a significant material with a diverse range of uses due to their unique combination of properties, including ultra-low density, adjustable porous structure, and exceptional mechanical characteristics. Synthetic polymer-based foams, which may be either biodegradable or non-biodegradable, dominate the global market. Non-biodegradable foams pose a significant environmental issue and have become a major challenge for waste management. Researchers have become interested in foams produced from renewable resources, particularly solid foams made from plant polymers [1,2,3].

Pulse proteins, such as those obtained from lentils, chickpeas, and peas have been studied for their many nutritional, functional, and structural properties. They have the ability to foam, and become foam stabilizers under varying conditions, refs. [4,5,6,7,8] demonstrating their versatility and potential for use in several food and non-food applications. However, their use for creating solid porous matrices or solid biofoams has only been receiving attention in recent years [9,10,11].

The creation of solid foams has some challenges that successful application depends on: (i) control of the structural properties of the liquid foam (bubble size distribution, pore opening, foam density, etc.), (ii) preservation of the liquid foam structure throughout the process, and (iii) the time scales pairing between the stability of the liquid foam and the solidification [12]. One major drawback, rooted in the internal structure of the biofoam, might be the structure collapse due to its weak microstructure.

The incorporation of natural fibers such as cellulose fibrils (CF), which have demonstrated an ability to improve the microstructure of polymer composites [13] might help overcome this issue. They can act by reinforcing the biofoam structure in order to endure certain drying processes. Some polysaccharide properties, such as the elastic modulus (134 to 143 GPa), and an entangled web-like structure, make them very attractive to produce structurally reinforced biofoams with diverse porous structures that will be able to withstand drying processes [10,14,15,16,17].

As the incorporation of CF might increase the viscosity of the liquid foam phase, regulating the viscosity becomes a critical parameter to control, in order to ensure a good foaming capacity of the pulse protein suspension while ensuring that the liquid foam remains to preserve the foam structure through the process, and the foam has enough stability and strength to go through the drying process.

To obtain solid foams from liquid foams, there are three steps to follow: (i) generate liquid foams, (ii) obtain enough foam stability during, and after foam generation to maintain the foam structure, then (iii) transform the liquid foam into a solid foam, typically done via polymerization (monomer) or cross-linking (polymer), which in turn results in the desired polymer foam after simple purification and/or drying steps [18]. After that, the solid foam needs to be characterized, as the structure-property relations are of particular interest and can be associated with the initial structural properties of the liquid foam [19].

Several processing methodologies have been reported in the literature to obtain biodegradable foams. Some of these methodologies are: supercritical carbon dioxide drying, extrusion/baking, and freeze-drying [9,10,11,14]. In addition, cellulose has already been utilized for producing porous materials [15,16,20]. Also, other compounds, such as starch, sunflower oil, gelatin, alginate, etc. have been mixed with cellulose in order to create new biomaterials [20,21,22,23]. However, these authors have not found studies involving pulse proteins and cellulose utilized for producing porous materials via freeze-drying yet.

In this research, biopolymer suspensions from lentil proteins are used as the continuous phase of a liquid foam system, which will be mixed with CF in order to study the effects of cellulose fiber on liquid lentil protein foam viscosity and foaming properties at different pH and cellulose concentration values. Furthermore, lentil protein foams were converted into solid foams by a convenient drying process. Moreover, their microstructure, some of their physical properties, and their morphology were assessed.

## 2. Experimental Methods and Materials

Valida-S^®^ (Cellulose fibrils) was kindly supplied by Sappi Global (Sappi Europe/Sappi Netherlands Services BV, Maastrich, The Netherlands). Lentil protein concentrate (85% protein) was obtained according to our previous work [24]. All other chemical reagents were purchased from Sigma-Aldrich (Santiago, Chile).

### 2.1. Preparation of Blended Lentil Protein/CF Foams

Lentil protein/CF foams were prepared according to Huang et al. (2018), with slight modifications [25]. Briefly, protein extract samples (10 mg/mL) were dispersed in 30 mL of deionized water adjusted to pH 3.0, 5.0, or 7.0 using either 0.5 mol/L NaOH or 0.5 mol/L HCl solutions. Each solution was mixed with a CF solution at 3 different concentration values (0.0, 0.1, and 0.5% *w*/*v*) and the pH value of the blended solutions were readjusted after this step. Higher values of CF concentration were too viscous to work with. The lentil protein-CF solutions were foamed with a homogenizer (Daihan Scientific, HG 15D, Wonju, Kangwon-do, Republic of Korea) for 2 min at speed 6. Samples were labeled according to pH and CF concentration as LP3, LP5, and LP7 for samples without CF and, LP (pH value)-0.1, and LP (pH value)-0.5, for samples with 0.1% or 0.5% CF, respectively. For example, samples at pH3 and 0.5% CF, were labeled as LP3-0.5.

### 2.2. Wet Foam Properties

Foaming properties

The foaming capacity (FC) was calculated as [5]:

(1) $$FC\left(\%\right)=\frac{\left(V_{f1}-V_{f0}\right)}{V_{f0}}\times 100$$

where V*_f_*_0_ and V*_f_*_1_ represent the volume of the blended lentil protein/CF solution and the formed foams after homogenization, respectively.

Foam stability (FS) was determined as [5]:

(2) $$FS\left(\%\right)=\frac{\left(V_{f2}\right)}{V_{f1}}\times 100$$

where V*_f_*_2_ is the volume of foam that remained after standing for 0.5 h at room temperature expressed as a percentage of the initial foam volume.

### 2.3. Shear Rheology Measurement

The rheological studies were conducted according to our previous study [5]. Preliminary dynamic oscillatory trials were conducted for the selection of suitable geometry to reduce foam slippage on the sensor’s surfaces, and the determination of appropriate gap size to prevent crushing and destruction of gas bubbles (1 mm). The geometry of the sensor and the size of the measurement gap were empirically selected using 95% reproducibility of the results as a criterion. Then, the viscoelastic properties of the protein-polysaccharide foams were measured as a function of time. Coalescence of droplets were not considered in the rheology measurements. Samples were analyzed using a controlled stress rheometer (Physica MCR300, Anton Paar, Graz, Germany) fitted with a cone and plate attachment with a diameter of 50 mm and angle of 2. Strain sweep tests with strain amplitudes ranging from 0.01% to 100% were performed at 0.6283 rad/s in order to establish the linear viscoelastic range. Based on these results, frequency sweep measurements (0.1–100 rad/s) were carried out at a strain amplitude of 1%; a value smaller than the critical value for linear viscoelasticity. Oscillatory measurements were performed in the linear region at a frequency of 0.6283 rad/s and strain of 1%, and the storage modulus G′ and loss modulus G″. In addition, shear rate-shear stress and shear rate-apparent viscosity data were collected as the shear rate was increased linearly between 1 and 100 s^−1^ over a total run time of 10 min. During the analysis, the sample was kept at 10 °C.

### 2.4. Solid Foam Preparation

Solid foam preparation was prepared according to Huang et al. (2018) [25]. Briefly, a 30 mL blended solution was foamed in a circular mold. The foam was neutralized with the atmosphere of liquid ammonia deposited in a desiccator for 12 h and then freeze-dried up to 24 h (LIOBRAS, L-108, São Carlos, Brazil). The resultant solid foams were labeled as their original LP-CF solutions.

### 2.5. Solid Foam Characterization

The volume of the dried foams was measured with a caliper. The density (d*_p_*) was estimated from the mass of the added components [22].

Water uptake capacity (WUR, also called swelling ratio) and solubility in water was determined gravimetrically according to the Cobb method, as previously mentioned in Zhang et al. (2011) and Chen et al. studies (2011) [15,26]. First, samples of determined size were weighed and soaked in 50 mL of distilled water for 30 min. After removing the excess water using tissue paper, the samples were weighed again. The quantity of adsorbed water was calculated as the weight difference, calculated as the mass of absorbed water per mass of the original sample, and expressed as a percentage value (ABNT NBR NM ISO 535, 1999) [26]. The reported values are the means of five measurements for each formulation. Solubility determination was determined gravimetrically as well, by drying swelled samples until reaching a constant weight in a ventilated oven at 105 °C. Both assays were performed at least in triplicate [20,21]. The water sorption capacity (WS) of samples at high relative humidity was also evaluated by a weight gain kinetics study over 4 days in a controlled atmosphere. All samples were placed in desiccators at 75% and 98% of relative humidity (RH), until the equilibrium was reached. Afterwards, the sample’s equilibrium moistures were determined by the gravimetric method drying until constant weight in an oven at 105 °C [20,21].

Foam morphology was observed by micrographs of the solid foam samples with a Hitachi X-650 scanning electron microscope (SEM, Hitachi, Ibaraki, Japan) at an acceleration voltage of 6 kV. Solid foams were sputtered with gold for 2 min before SEM observation.

### 2.6. Statistical Analysis

All samples were tested in triplicate and results are presented as mean ± SD. One-way or two-way analysis of variance (ANOVA) was carried out using R Statistical Software (v4.1.2) [27], and statistical differences among sample means were determined using Tukey’s test at a 95% confidence level.

## 3. Results and Discussion

### 3.1. Foaming Properties

The foaming capacity (FC) as a function of pH is presented in Table 1. The FC values for LP-0.1CF and LP-0.5CF are significantly lower than LP at any pH value (p < 0.01); then, the presence of CF at any concentration reduced the FC of lentil protein. Cellulose addition makes the liquid phase more viscous, hindering the expansion of gas bubbles, which resulted in lower foaming capacity [22,23]. Also in Table 1, the results for foaming stability (FS) are presented. On the contrary to FC, all samples containing CF demonstrated higher FS values than samples without cellulose regardless of pH value or CF concentration, though only values at pH 3.0 and 5.0 presented significant differences (p < 0.01). The FS values ranged from 51.6% for the sample LP5 to 77% for the sample LP3-0.1, indicating FS was improved by the presence of CF. Improving FS of LP-CF mixtures might be related to two phenomena. As CF concentration increased, so did the viscosity of the mixture, which might help to preserve the stability of the samples. Another factor is the interaction between lentil protein and cellulose. The surface charge of LP moves from positive to negative values as the pH increases, while CF has a negative surface charge along the pH spectrum (Table 2). Typically, coacervates are created between proteins and anionic polysaccharides below the protein’s isoelectric point. This is due to the attractive forces between the negatively charged groups (COO^-^) on the polysaccharide chains and the positively charged groups (NH_3_^+^) on the protein chains [28,29]. Coacervates might help to stabilize the foams by thickening the interfacial layer and retarding coalescence due to the formation of an electrostatically cross-linked gel-like interfacial network, thus influencing foaming properties at pH 3.0 and stabilizing the foams against collapse [30]. On the other hand, a pH of 7.0 leads to the segregation of LP and CF mixtures, which is caused by the repulsion of electrostatic interactions and differences in solvent affinities. The degree of incompatibility between the polymers determines whether they will separate, resulting in a water-in-water emulsion-like system composed of “droplets” that are rich in polysaccharide and surrounded by a protein-rich continuous phase, or vice versa, depending on the ratio of the biopolymers. This phenomenon has been studied by several researchers including Asghari et al. (2016), Ganzevles et al. (2007); Perez et al. (2010), and Zhang et al. (2021) [28,30,31,32].

### 3.2. Shear Rheology Measurement

**Figure 1:**
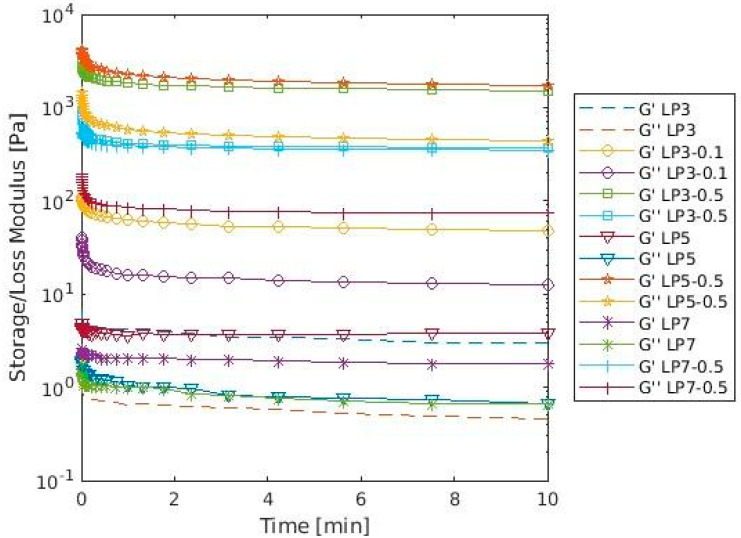
Storage (G′) and loss (G″) modulus of different combinations of LP-CF foams as a function of time.

**Figure 2:**
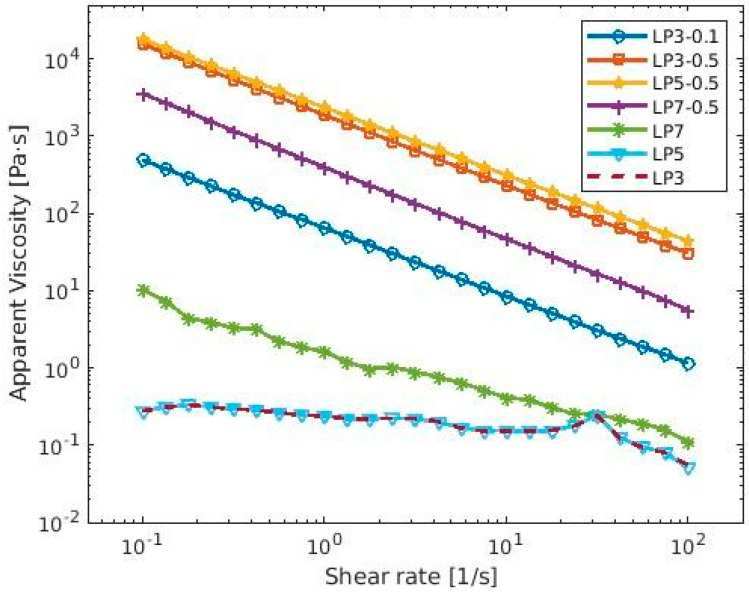
Apparent viscosity of lentil protein-CF foams as a function of shear rate at different pH values.

The surface properties of CF and proteins influence interfacial and rheological properties [33]. All LP-CF foams showed higher storage modulus (G′) values than LP foams (Figure 1). Also, foams with higher CF concentration (0.5%) had higher G′ and G″ values than foam with 0.1%CF. This can be attributed to an increase in the intermolecular protein-cellulose interactions at pH 3.0 or protein-protein interactions at pH 5.0 and 7.0 at the surface layer of adsorbed proteins. Differences in z-potential values suggest electrostatic interaction between LP and CF at pH 3.0, enhancing interface structure, which might explain higher G′ values when compared to lentil protein foam alone. As for foams at pH 5.0 and 7.0, electrostatic repulsion between LP and CF (Table 2), might favor protein-protein intermolecular bonds and protein coagulation [34]. Those interactions still remain to be identified, and they should be studied in the future to better understand foam rheological properties. Regarding G″, these values remained lower than G′ throughout the entire testing period, which suggests that the LP-CF mixture formed an elastic network at the air-water interface across all tested pH values. As G′ value is the expression of resistance to deformation and G″ measures energy lost once the stress is removed, higher values of G′ are related to elastic behavior. Also, is the interface where rheology phenomena occur, meaning, surface energy is stored or loosened. Then, the elastic behavior should be the result of interface modification [35]. This elastic film might significantly contribute to foam stability by resisting deformation, which would agree with higher FS values in all LP-CF foams (Table 1). As for apparent viscosity results (Figure 2), all LP-CF foams showed higher values than LP foams and a shear thinning behavior, which has been previously observed in other protein-polysaccharide foams that contains cellulose fibers [5,36]. Disaggregation of aggregated droplets during shearing is the main cause of the shear thinning phenomenon in foams. Also, the alignment of the protein chains in the direction of the shear force or the mechanical damage during the measurement might be responsible to a lesser extent for foam disruption, if the protein chains are long or the shear rate is high [37]. In addition, it is highly possible that the viscosity of CF (0.5–1.04 Pa s) added to intermolecular interactions is responsible for higher apparent viscosity values of LP5-0.5 and LP3-0.5, i.e., protein aggregation produced at pH 5.0 (due to proximity to isoelectric point) and protein-cellulose coacervation at pH 3.0 (positive and negative molecules charge) [23].

### 3.3. Solid Foam Characterization

Table 3 shows the characterization parameters of obtained solid foams. No significant difference was found for the measured parameters, except for WUR and water sorption at 98% R.H. LP3-0.1 foam had a significantly lower WUR value than other foams. According to Zhang et al. (2011) [26], WUR is related to pH because of changes in the charges of polymers. In solution, typically an anionic polymer (as CF in this study) is protonated at a low pH but ionized at a high pH. Thus, under low pH, most of the carboxylate anions are protonated, the anion–anion repulsive forces are eliminated and, consequently, the water absorbency declines. Also, for LP3-0.1CF concentration of CF is lower than other foams, which might contribute to the low value of WUR. Concerning moisture and water sorption, both parameters are related to the hydrophilic character of foam compounds [21]. Moisture values are similar to other studies [10,11,21]. However, water sorption results were more limited than other biofoams [11,16,23]. This behavior might be explained by the different chemical compositions of biofoams obtained in this study. It seems that CF is less hydrophilic than filler compounds utilized by Versino et al. (2021) [21] and Cervin et al. (2013) [16]. Differences with Ago et al. (2016) study [11], which used lignin, might be explained based on starch presence. Starch water absorption capacity is higher than cellulose and lentil protein, thus, it is probably responsible for this difference [5,38]. Also, reduced values of water sorption capacity might be explained by the interaction between cellulose and hydrophilic sites of LP, which substitutes the LP-water interaction [39,40]. As water sorption also depends on morphology, specific surface area, density, etc., so these results correlate well with morphology (see discussion in Section 3.4) and density (Table 3). Regarding density, there is no significant difference between values. Also, our findings are in line with densities reported by other authors working on similar solid foams [21,23]. Finally, solubility values are high for all the samples. Even though differences are not significant, LP3-0.1 was remarkably high (90.8%) in comparison to other values, and the sample was almost completely solubilized during testing. It is probable that the open pore structure of LP3-0.1 makes it easy for water to fill the foam and facilitates the solubilization mechanism as well [21,41]. This also relates well with the results for the LP7-0.5 sample.

### 3.4. Solid Foams Morphology

**Figure 3:**
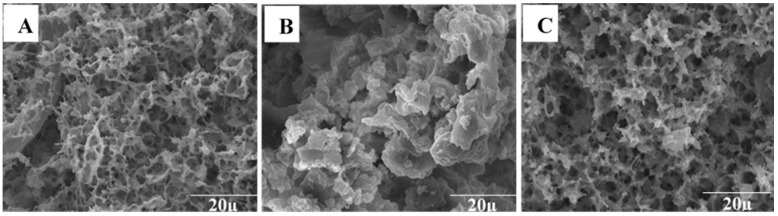
Lentil protein foam micrographs: pH 3.0 (**A**), pH 5.0 (**B**), and pH 7.0 (**C**).

**Figure 4:**
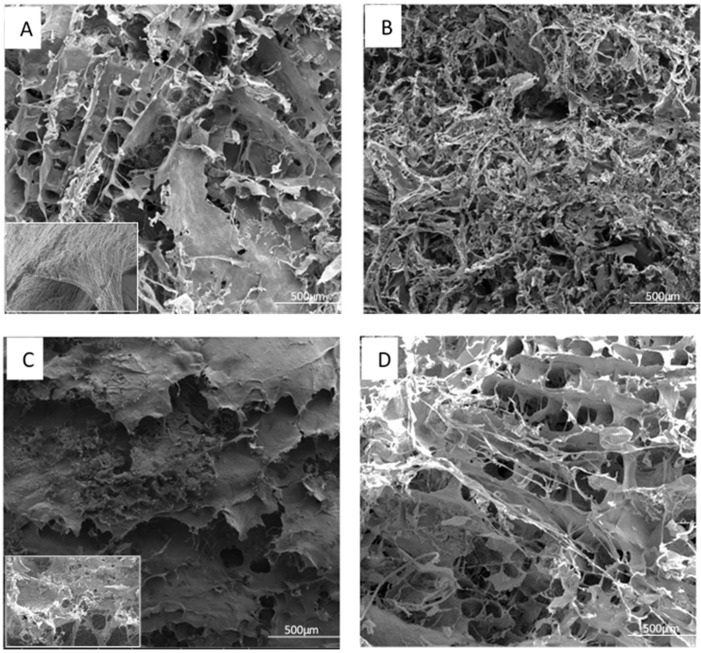
LP-CF SEM micrographs of foams produced at different pH and CF concentrations: LP3-0.1 insert at 10,000× (**A**), LP3-0.5 (**B**), LP5-0.5, insert at 5000× (**C**), and LP7-0.5 (**D**).

Observation of lentil protein foam using scanning electron microscopy (SEM) under acidic conditions (pH 3.0) revealed a less densely packed structure, as depicted in Figure 3A. On the other hand, when the foam was examined at a pH of 5.0 (Figure 3B), it exhibited a particle-like morphology characteristic of protein networks that form near the protein’s isoelectric point (pI). This network consisted of randomly aggregated protein particles, resulting in a thick and dense structure. The foam prepared at a neutral pH displayed a structure similar to the one prepared at pH 3.0 (Figure 3C). In comparison, Figure 4 shows the internal structure and morphology of the polymeric foam obtained from LP-CF mixtures. It can be seen from these images how the presence of CF modifies the microstructure. LP3-0.1 and LP5-0.5 foams (Figure 4A,D) have open pore structures and pore shapes that are semi-spherical. It seems that CF fibers extend along forming layers, apparently giving support to the microstructure. However, the structure is irregular. LP3-0.5 and LP5-0.5 (Figure 4B,C) have highly irregular pore sizes and shapes throughout the samples. They also exhibit a much denser structure than LP3-0.1 and LP7-0.5, which is probably partially responsible for some of the foam characteristics (Table 3), for example, density and solubility [20,25]. Also, the apparent viscosity shown in Figure 1, seems to be related to the final microstructure of the foams, which makes sense, since several studies have demonstrated that viscosity helps preserve the wet foam structure during freezing and lyophilization processes [42]. Though, these apparently higher viscosity values diminished their other characteristics, such as foaming capacity, yet make denser structures instead.

## 4. Conclusions

The foam properties of LP-CF mixtures were significantly affected by changes in CF concentration, especially because of higher viscosity values. The foam’s average lifespan tended to increase at any pH value when CF concentration rose.

The microstructure of the foams suggests a logical connection with apparent viscosity results and it is reasonable to consider that viscosity plays a role in maintaining the integrity of the wet foam structure during freezing and lyophilization procedures. However, these elevated apparent viscosity values may result in a decrease in other properties such as foaming capacity, and an increase in the formation of denser structures.

The internal structure and morphology of the polymeric foam obtained from mixtures of LP-CF showed that some of the foams possess a sponge-like structure, though with open pores and semi-spherical pore shapes. In these foams, CF fibers appear to be extending and forming layers, providing support to the microstructure. However, the structure itself is irregular. On the other hand, LP3-0.5 and LP5-0.5 exhibited a highly non-uniform pore size throughout the samples, accompanied by irregular pore shapes. These foams also display a denser structure compared to LP3-0.1 and LP7-0.5, which likely contributes to determining different foam characteristics, such as density and solubility, between the samples.

These results help understand the behavior of lentil protein and cellulose fibrils mixture, though other important characteristics remain to be determined, such as mechanical properties, biodegradability, and hydrophobicity, in order to fully comprehend the potential use of these foams.

## Figures and Tables

**Table 1:** Wet Foam Properties at Different pH Values.

	FC (%)			FS (%)		
Samples	pH 3	pH 5	pH 7	pH 3	pH 5	pH 7
LP	113.3 ± 6 ^a,x^	133.2 ± 6 ^a,x^	100.0 ± 5 ^a,x^	56.0 ± 3 ^a,x^	51.6 ± 5 ^a,y^	60.3 ± 6 ^a,z^
LP-0.1CF	73.1 ± 6 ^b,x^	87.7 ± 6 ^b,x^	93.3 ± 6 ^b,x^	77.0 ± 5 ^b,x^	57.4 ± 3 ^b,y^	62.1 ± 4 ^a,z^
LP-0.5CF	93.3 ± 6 ^b,x^	87.6 ± 8 ^b,x^	86.7 ± 4 ^b,x^	58.9 ± 3 ^ac,x^	68.3 ± 4 ^ac,y^	68.2 ± 4 ^a,z^

Different letters within the same column indicate significant differences among samples (p < 0.05) a, b, c: Significant difference between samples at fixed pH. x, y, z: significant differences between pH values for the same sample.

**Table 2:** Ζ-Potential Values (mV) for Lentil Protein (LP) and Cellulose Fibrillated (CF) at Different pH Values.

	pH 3	pH 5	pH 7
LP	14.6 ± 0.5	−20.6 ± 0.3	−24.2 ± 0.2
CF	−26.2 ± 2.4	−26.3 ± 1.3	−22.9 ± 1.6

**Table 3:** Solid Foam Properties.

Sample	Density (g/cm^3^)		WUR (%)		Solubility (%)		Moisture (%)		WS-75%R.H (g_H2O_/g_DB_)		WS-98%R.H (g_H2O_/g_DB_)	
LP7-0.5	0.057 ^a^	±0.020	423 ^a^	±72	78.0 ^a^	±12.6	16.9 ^a^	±2.2	0.04 ^a^	±0.01	0.40 ^a^	±0.07
LP5-0.5	0.065 ^a^	±0.011	554 ^ab^	±86	65.5 ^a^	±15.7	20.4 ^a^	±6.5	0.02 ^a^	±0.01	0.39 ^ac^	±0.05
LP3-0.1	0.053 ^a^	±0.011	256 ^ac^	±75	90.8 ^a^	±3.9	15.7 ^a^	±1.1	0.03 ^a^	±0.01	0.24 ^acd^	±0.04
LP3-0.5	0.055 ^a^	±0.010	667 ^abd^	±51	68.5 ^a^	±3.2	14.1 ^a^	±6.0	0.04 ^a^	±0.01	0.10 ^b^	±0.00

Different letters within the same column indicate significant differences among samples (p < 0.001). DB dry basis.

## Data Availability

The data presented in this study are available on request from the corresponding author. The data are not publicly available due to privacy restrictions.
